# Conserved Overlapping Gene Arrangement, Restricted Expression, and Biochemical Activities of DNA Polymerase ν (POLN)*[Fn FN2]

**DOI:** 10.1074/jbc.M115.677419

**Published:** 2015-08-12

**Authors:** Kei-ichi Takata, Junya Tomida, Shelley Reh, Lisa M. Swanhart, Minoru Takata, Neil A. Hukriede, Richard D. Wood

**Affiliations:** From the ‡ Department of Epigenetics and Molecular Carcinogenesis, University of Texas M.D. Anderson Cancer Center, Smithville, Texas 78957,; the § University of Texas Graduate School of Biomedical Sciences at Houston, Houston, Texas 77030,; the ¶ Department of Developmental Biology, University of Pittsburgh School of Medicine, Pittsburgh, Pennsylvania 15213, and; the ‖ Laboratory of DNA Damage Signaling, Department of Late Effects Studies, Radiation Biology Center, Kyoto University, Kyoto 606-8501, Japan

**Keywords:** alternative splicing, BRCA1, DNA damage, DNA polymerase, zebrafish

## Abstract

DNA polymerase ν (POLN) is one of 16 DNA polymerases encoded in vertebrate genomes. It is important to determine its gene expression patterns, biological roles, and biochemical activities. By quantitative analysis of mRNA expression, we found that *POLN* from the zebrafish *Danio rerio* is expressed predominantly in testis. *POLN* is not detectably expressed in zebrafish embryos or in mouse embryonic stem cells. Consistent with this, injection of *POLN*-specific morpholino antisense oligonucleotides did not interfere with zebrafish embryonic development. Analysis of transcripts revealed that vertebrate *POLN* has an unusual gene expression arrangement, sharing a first exon with *HAUS3,* the gene encoding augmin-like complex subunit 3. *HAUS3* is broadly expressed in embryonic and adult tissues, in contrast to *POLN*. Differential expression of *POLN* and *HAUS3* appears to arise by alternate splicing of transcripts in mammalian cells and zebrafish. When POLN was ectopically overexpressed in human cells, it specifically coimmunoprecipitated with the homologous recombination factors BRCA1 and FANCJ, but not with previously suggested interaction partners (HELQ and members of the Fanconi anemia core complex). Purified zebrafish POLN protein is capable of thymine glycol bypass and strand displacement, with activity dependent on a basic amino acid residue known to stabilize the primer-template. These properties are conserved with the human enzyme. Although the physiological function of pol ν remains to be clarified, this study uncovers distinctive aspects of its expression control and evolutionarily conserved properties of this DNA polymerase.

## Teaser

**Background:** The biological function of DNA polymerase ν (POLN) is unknown.

**Results:** Vertebrate *POLN* genes are predominantly expressed in testis, share a first exon with *HAUS3*, and encode proteins with strand displacement and damage bypass activity.

**Conclusion:** These properties indicate a specific POLN function in testis.

**Significance:** Conserved biochemical activities, expression patterns, and protein interactions suggest a restricted function of POLN in DNA processing.

## Introduction

It is remarkable that the genomes of higher eukaryotes encode so many different DNA polymerases. Each of these enzymes is specialized to operate in some aspect of DNA replication, pathways of DNA repair, diversification of antibody genes, or in translesion DNA synthesis. In human cells, defects or mutations in DNA polymerases increase predisposition to various cancers (1).

**FIGURE 1.**
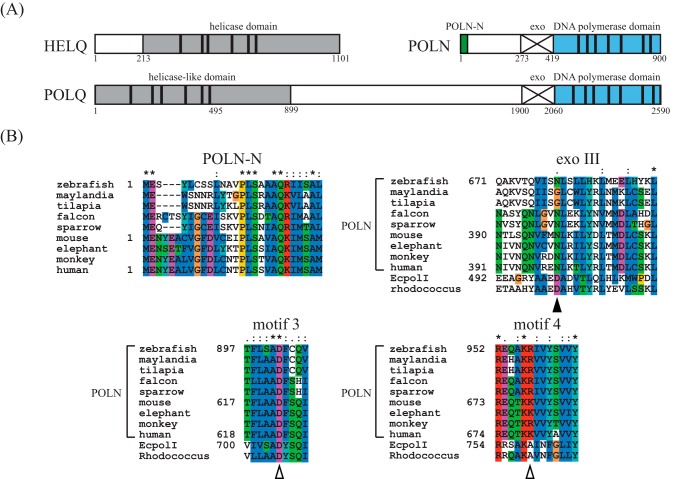
**Conserved features of DNA polymerase ν (POLN).** *A,* domains of human POLN, POLQ, and HELQ. Defined motifs are shown by *vertical black stripes. B,* sequence alignment of POLN-N, exoIII, motif 3, and motif 4 of POLN from three fish (zebrafish, maylandia, and tilapia), two birds (falcon and sparrow), four mammals (mouse, elephant, monkey, and human), and prokaryotic A-family DNA polymerases, *E. coli* DNA polymerase I (*EcpolI*) and *Rhodococcus erythropolis* DNA polymerase I (*Rhodococcus*). Residues are colored in similarity groups as follows: {K, R, H}, {D, E}, {I, L, V, M}, {F, Y, W}, {Q, N}, {G, A}, {S, T}, {P}, and {C}. Perfectly conserved residues are denoted by *, and highly or relatively conserved residues are denoted by *colons* and *periods*, respectively. The *open arrowheads* show the residues Asp-902 and Arg-957 of zebrafish POLN substituted in this study. The *closed arrowhead* shows an Asp residue essential for 3′–5′-exonuclease (*exo*) activity, which is absent in POLN. The sequence alignment was carried out using the Clustal X program.

Human DNA polymerase ν is encoded by the *POLN* gene. It is a member of the DNA polymerase A-family (2–4). The DNA polymerase domain of POLN is related to that of mammalian POLQ/*Drosophila* Mus308 (Fig. 1*A*) (2, 5). Mus308 and POLQ participate in a pathway of DNA double strand break repair by alternative end joining (6, 7). Defects in Mus308 or POLQ confer hypersensitivity to various DNA-damaging agents (7–9). Both *mus308* and *POLQ* also encode an N-terminal helicase-like domain (10, 11). A third member of this gene family, *HELQ*, encodes a helicase domain similar to that of POLQ/Mus308 (Fig. 1*A*) (12). HELQ interacts with ataxia telangiectasia and Rad3-related (ATR) and with homologous recombination-related RAD51 paralogs and participates in DNA cross-link resistance in human cells (13–15).

The function of POLN is currently uncertain, and several roles have been suggested. It has been reported that siRNA mediated knockdown of *POLN*-sensitized human cells to DNA cross-linking agents (16, 17). However, *POLN*^−/−^ chicken DT40 cells were not sensitive to mitomycin C, cisplatin, or camptothecin (18, 19). Instead, it was proposed that POLN functions in homologous recombination reactions in chicken cells, leading to immunoglobulin V gene diversification by gene conversion (19). In mouse tissues, expression of *Poln* can be detected by northern blotting only in the testis (2). It is uncertain whether *POLN* is significantly expressed in other tissues or during development and whether the gene is essential for embryogenesis.

Previous studies of recombinant human POLN also hint at diverse functions for the protein by revealing several unique biochemical properties. The human enzyme has efficient strand displacement activity and low fidelity steady-state incorporation of T opposite template G (3, 20, 21). *In vitro*, POLN is proficient in the accurate bypass of major groove DNA lesions including a Tg^3^ and major groove DNA-peptide and DNA-DNA cross-links (3, 22). POLN cannot bypass a number of other DNA modifications, including an abasic (AP) site, a cisplatin-induced intrastrand d(GpG) cross-link, a cyclobutane pyrimidine dimer, a 6-4 photoproduct, or minor groove DNA peptide or DNA-DNA cross-links (3, 22). We found that evolutionarily conserved residues in the O-helix of POLN are critical for the low fidelity and bypass activity of human POLN (4). However, when the O-helix of KlenTaq, a high fidelity A-family DNA polymerase, was replaced with the corresponding sequence from POLN, the fidelity of the mutant KlenTaq was higher than that of POLN and similar to that of the parental wild-type KlenTaq (23). The result suggested that the O-helix of POLN is not the only determinant critical for unique properties of human POLN. It is important to examine the fidelity properties of POLN in other species.

The *POLN* gene is present in the genomes of deuterostomes, including vertebrates. Here, we describe the restricted expression of *POLN* in the zebrafish *Danio rerio*. We report the discovery of an unusual overlapping relationship between the *POLN* and *HAUS3* genes in vertebrates. These two genes share the same first exon, but they have very different expression patterns. We also found that ectopically expressed POLN can interact with protein components of the DNA recombination machinery.

## Experimental Procedures

### 

#### 

##### Isolation of the Zebrafish DNA Polymerase N (DrPOLN) Gene

Searches of the Zebrafish Model Organism Database revealed a zebrafish chromosome 7 genomic DNA sequence, NW_001879254 (NCBI accession number), which encodes several exons homologous to the human POLN polymerase domain. From this sequence, primers were designed to clone the zebrafish coding sequence by 3′- and 5′-rapid amplification of cDNA ends (BD Biosciences SMART RACE cDNA amplification kit). Total RNA was prepared from zebrafish testes using TRIzol (Life Technologies, Inc.). The full-length cDNA was cloned into plasmid pCR4-TOPO (Invitrogen), and the *DrPOLN* cDNA sequence was submitted to NCBI, accession number DQ630550.

##### Construction of DrPOLN Derivatives

We were unable to express full-length DrPOLN in *Escherichia coli*, but we succeeded with a construct beginning after the ninth Met, encoding amino acids 276–1146. This was amplified by PCR from the full-length *DrPOLN* plasmid using the primers 5′-CACCGAAAACTCTCCAGATGCCAAAAGATG-3′ (for the 5′ end) and 5′-ATATATGAATTCCTACTTGTCGTCATCGTCTTTGTAGTCGGCAGAAGTTGCTGTAGCGGTG-3′ (for the 3′ end) and cloned into plasmid pENTR/D-TOPO (Invitrogen). After DNA sequencing, the cDNA was transferred into plasmid pDEST17 (Invitrogen) resulting in a protein tagged with six His residues at the N terminus (contributed by the pDEST17 vector), and a FLAG tag at the C terminus. Primers containing DrPOLN point mutations (altered DNA sequences areunderlined) were synthesized as follows: 5′-CTTTCCTCTCTGCAGCTTTCTGTCAGGTGGAG-3′ and 5′-CTCCACCTGACAGAAAGCTGCAGAGAGGAAAG-3′ (for D902A); 5′-CAGAGAGCAGGCCAAGGCGATCGTCTACTCTGTG-3′ and 5′-CACAGAGTAGACGATCGCCTTGGCCTGCTCTCTG-3′ (for R957A). Site-directed mutagenesis was performed by using the QuikChange II site-directed mutagenesis kit (Stratagene). To generate D902A and R957A mutations, the pDEST17 vector carrying *DrPOLN* (amino acids 276–1146) was used as a template. Recombinant POLN derivatives were bacterially expressed and purified as reported (3, 4). These proteins were concentrated by NANOSEP 30K (PALL) and stored in buffer (50 mm sodium phosphate, pH 7.0, 300 mm NaCl, 10% glycerol, and 0.01% Nonidet P-40). Soluble full-length DrPOLN could not be purified under these expression conditions. Human POLN and RB69 gp43 were purified as reported (3, 24) and were used as controls.

##### Oligonucleotide Substrates

Primer oligonucleotides were purchased from Bio-Synthesis or Sigma Genosys, purified by gel extraction, and 5′-labeled using [γ-^32^P]dATP with polynucleotide kinase. Oligonucleotides containing a Tg were synthesized as described (3). Substrates for DNA polymerase assays were constructed by annealing 5′-^32^P-labeled CACTGACTGTATGATG-3′ primer to 3′-GTGACTGACATACTAC*X*TCTACGACTGCTC-5′ template. The first template base (denoted by *X*) was T, G, or Tg. To form the nicked substrate, 5′-AAGATGCTGACGAG was additionally annealed to a template where *X* = T.

##### DNA Polymerase Assays

A 5′-^32^P-labeled 16-mer primer and a 30-mer template (sequences given above) were annealed at a molar ratio of 1:1 to detect DNA polymerase activity. 5′-^32^P-Labeled 16-mer primer, a downstream oligomer (5′-AAGATGCTGACGAG-3′), and the 30-mer template at a molar ratio of 1:5:2 were used as a nicked substrate. Primer-templates were heated for 5 min at 65 °C and cooled down slowly for annealing as follows: 37 °C for 30 min, 25 °C for 20 min, and 4 °C for 20 min. Reaction mixtures (10 μl) contained 20 mm Tris-HCl, pH 8.8 (unless otherwise indicated), 4% glycerol, 2 mm dithiothreitol (DTT), 80 μg/ml bovine serum albumin (BSA), 8 mm magnesium acetate, 30 nm of the primer-template, 100 μm of each dNTP, and the indicated amount of DrPOLN derivatives. RB69 gp43 reaction mixtures (10 μl) contained 10 mm Tris-HCl, pH 7.9, 50 mm NaCl, 1 mm DTT, 200 μg/ml BSA, 10 mm MgCl_2_, and 100 μm of each dNTP. After the addition of the enzymes, the reaction mixture was incubated at 37 °C for 10 min; reactions were terminated by adding 10 μl of formamide stop buffer (98% deionized formamide, 0.025% xylene cyanol, 0.025% bromphenol blue, and 20 mm EDTA) and boiling at 95 °C for 3 min. Products were electrophoresed on a denaturing 20% polyacrylamide, 7 m urea gel, exposed to BioMax MR film, and analyzed with a Fuji FLA3000 PhosphorImager. For translesion synthesis, the same amounts of templates containing specific lesions were used.

##### Translesion DNA Synthesis Bypass Efficiency Reactions

The assays were performed as reported (3, 4). The 5′-^32^P-labeled primers (5′-CACTGACTGTATGA-3′ or 5′-CACTGACTGTATGAT-3′) in Fig. 7 are similar but are two or one nucleotide shorter than the primer used for DNA polymerase assays in Fig. 6 (5′-CACTGACTGTATGATG-3′). The 5′-^32^P-labeled 14- or 15-mer primer and a 30-mer template (sequences given above) were annealed at a molar ratio of 1:1. Primer extension reactions with DrPOLN and R957A were as described above. 10 μl of reaction mixtures were incubated at 37 °C for 2, 4, and 6 min and diluted in 10 μl of formamide stop buffer. Products were heated at 95 °C for 3 min and separated on a denaturing 20% polyacrylamide, 7 m urea gel. Product bands were quantified by PhosphorImager, and the values were used to calculate the probability of termination of processive synthesis and the insertion efficiencies at each template nucleotide. The termination probability at any position (*N*) is defined as the band intensity at *N* divided by the total intensity for all bands ≥*N*. The insertion probability at any position (*N*) is defined as the intensity at bands ≥*N* divided by the intensity at bands ≥*N* − 1. The extension probability at any position (*N*) is defined as the band intensity ≥*N* + 1 divided by the intensity at bands ≥*N*. The bypass probability at position *N* is defined as the band density ≥*N* + 1 divided by the intensity of ≥*N*_1_. To detect the bypass efficiency, the bypass probability (damaged) is divided by the bypass probability (undamaged) as described (25). The values are averages from two independent experiments at reaction intervals from 2, 4, and 6 min.

##### Cloning of 5′-Untranslated Region (UTR) of Human DNA Polymerase N (HsPOLN)

The 5′UTR of *HsPOLN* was isolated from human testis total RNA (Clontech) using a SMART RACE cDNA amplification kit (BD Biosciences). The 5′UTR was cloned into pCR4-TOPO (Invitrogen) and sequenced.

##### Quantitative PCR (qPCR) Assay

Total RNA was extracted using TRIzol, and RNA integrity was assessed using the Agilent 2100 bioanalyzer (Agilent Technologies, Inc.). Total RNA (1 μg) was then used as template to synthesize cDNA with the High Capacity cDNA archive kit (Applied Biosystems). qPCR was then performed on the Applied Biosystems 7900HT fast real time PCR System (Applied Biosystems). Custom assays for zebrafish, *DrPOLN*, *DrPOLQ*, *DrHAUS3*, and *Dr*β-*ACTIN,* and *Homo sapiens, HsPOLN, HsPOLQ,* and *HsHAUS3,* were designed using FileBuilder 3.1 software (Applied Biosystems) and ordered from Applied Biosystems. TaqMan primer and probe sets for each gene are shown in Table 1. TaqMan primers and probe set for human *GAPDH* was purchased from Applied Biosystems. Triplicate qPCRs each containing cDNA representing 40 ng of reverse-transcribed total RNA were then assayed for transcript quantity with *Dr*β-*ACTIN* and *HsGAPDH* serving as endogenous controls to normalize input RNA levels. The absolute quantity of transcripts for the gene of interest in each sample was determined using the generated standard curves and the Sequence Detection Software version 2.2.2 (Applied Biosystems). Standard curves for each gene were determined using the following plasmids: pDEST17 carrying cDNA coding 276–1146 amino acids of *DrPOLN*; full-length open reading frame (ORF) of *DrHAUS3* cloned into pCR4; *DrPOLQ* clone (clone ID 8345083; Open Biosystems); full-length *HsPOLN* ORF cloned into pDEST17 (4); *HsPOLQ* inserted into pFASTBac (11); *HsHAUS3* clone (clone ID 3534250; Open Biosystems).

**TABLE 1:** **TaqMan primer and probe sets**

	TaqMan primer	TaqMan probe
*DrPOLN*	5′-TCAAGACGGCACCACACAA	5′-TTGGACTCAGAACAGAAACT
	5′-GATCAGCAGGCCACACACT	
*DrPOLQ*	5′-ACCCTGCTTCAGTTGATGATAACAT	5′-CAGGCCAAACAGATTTG
	5′-GCACCCATACCGTAGATTATTCCAT	
*DrHAUS3*	5′-TGGCTTATCCAGCGATTGAACA	5′-CACAGCCTCTGAGCTGC
	5′-TGCTCATCGGCGAGTAGTG	
*Dr*β*-ACTIN*	5′-CTGAATCCCAAAGCCAACAGAGA	5′-CATGATCTGTGTCATCTTC
	5′-GCCTGGATGGCCACATACAT	
*HsPOLN*	5′-ACTGATGGTTCCACCCAGCTA	5′-ACCAGACCCCCGTTTCT
	5′-GCGTTTTACTAACACCACAATTCCT	
*HsPOLQ*	5′-GGAGGTGGAGGTGATTCTGAAAA	5′-TCCGGGCACTTTTG
	5′-ACTGCTTCCTCTTCCTCATCCA	
*HsHAUS3*	5′-TCTCAACAGATAAATCCAAGGAATACCATTG	5′-CTCATAGGCTTTACCAAGTTT
	5′-AACAATTCTTTTTTCTTATTCTCTCCCTCCA	

##### Northern Hybridization

Total RNA was purified from zebrafish tissues using TRIzol. For isolation of poly(A)^+^ RNA from total RNA, an Oligotex Direct mRNA mini kit (Qiagen) was used. 2 μg of each poly(A)^+^ RNA was separated on a 1.0% formaldehyde-agarose gel with 1× MOPS buffer. After soaking in 50 mm sodium hydroxide for 25 min and twice in 200 mm sodium acetate, pH 4.0, for 20 min, the gel was transferred to a nylon membrane (BrightStar-Plus; Ambion) with 20× SSPE. The membrane was UV cross-linked, dried at 80 °C for 2 h, and stored at −20 °C. After prehybridization with ULTRAhyb (Ambion) containing 1 mg/ml torula yeast total RNA (Sigma), the filter was probed with ^32^P-labeled *DrPOLN* cDNA (DQ630550), *DrHAUS3* cDNA (BC124280), or *Dr*β-*ACTIN* cDNA (BC063950) at 42 °C for 16 h, followed by washing twice with 2× SSPE + 1% SDS at room temperature for 15 min and twice with 1× SSPE + 0.1% SDS at 50 °C for 20 min. Blots were exposed to Kodak X-Omat XAR film.

##### In Situ Hybridization

Zebrafish embryos were fixed in 4% formaldehyde in PBS and processed for whole mount *in situ* hybridization as described (26). pCR4 plasmid containing 895 bp of *POLN* cDNA, including 400 bp of its 3′-untranslated region, and pCR4 plasmid containing the full-length open reading frame of *HAUS3* were used as templates for *in vitro* transcription to obtain antisense and sense probes. Antisense RNA probes for *POLN* and *HAUS3* were made by digestion of the vectors with PmeI and transcription with T7 RNA polymerase, and sense probes were made by digestion of the same vectors with NotI and transcription with T3 RNA polymerase. Probe RNA was labeled with digoxigenin-dUTP using an RNA labeling kit (Roche Applied Science).

##### Zebrafish POLN Morpholino Knockdown

Zebrafish embryoswere obtained from in-crossing wild-type adults maintained at 28.5 °C. A complementary MO (5′-TGCAGAGGTAGCTCTCCATGTTCGT-3′) targeting the initiation codon of zebrafish POLN was obtained from GeneTools LLC. Embryos were injected at the one-cell stage with 2.5, 5, or 10 ng of POLN MO. The control group was mock-injected.

##### Reverse Transcriptase PCR (RT-PCR)

Total RNA was isolated from mouse R1 ES cells and FVB mouse testes. Human testis total RNA was purchased from Clontech. First-strand cDNA was synthesized from 1 μg of total RNA using the SuperScript III First-Strand Synthesis System (Invitrogen) with oligo(dT)_20_ primer and amplified using the following primers: human F1, 5′-TGCGAGAAGCAAGCGGAAC-3′; F2, 5′-TACACCGCTCTCCAGTGTTG-3′; F3, 5′-GTTTGAGGGCGTTGAAGATG-3′; F4, 5′-CAATGGACCTTTGCTCTAAACTG-3′; B1, 5′-CTGATGTTGAGCACAAATGTATGC-3′; B2, 5′-CCGTTCTCCTGCAACAAAAT-3′; β-actin (sense), 5′-GCTCGTCGTCGACAACGGCTC-3′; β-actin (antisense), 5′-CAAACATGATCTGGGTCATCTTCTC-3′; and mouse primer 1, 5′-AGCAGTGACTGCGGCTTTCCTG-3′; primer 2, 5′-ATGAGTTGTGGAAATGAGTTTG-3′; primer 3, 5′-TTATCTTTCCACTTGATGCAATTTATTAG-3′; primer 4, 5′-AAAATGGAAAATTATGAGGCATGTG-3′; primer 5, 5′-GGCTGAGCCCAGGATCTCCTG-3′; K*Rev3*-forward, 5′-GTGGCGGCGGCGGCGAACATG-3′; K*Rev3*-reverse, 5′-GGCATTCCTGATACTAATGACAC-3′; 5′Mm*PolL*, 5′-ATGGACCCTCAGGGCATCGTG-3′, and 3′*MmPolL*, 5′-TCACCAGTCCCGTTCAGCTGG-3′. F1 can be annealed to the 5′UTR of *HsPOLN and HsHAUS3*; F2, F4, and B2 are for ORF of *HsPOLN*; F3 and B1 are for ORF of *HsHAUS3*. Primer 1 can be annealed to the 5′UTR of *MmPoln* and Mm*Haus3*; primers 2 and 3 are for ORF of Mm*Haus3*; primers 4 and 5 are for ORF of *MmPoln*. As controls K*Rev3*-forward and K*Rev3*-reverse, which can amplify two alternative splicing variants of *MmRev3L* (27), 5′*MmPolL* and 3′*MmPolL*, which can amplify ORF of *MmPolL*, β-actin (sense and anti sense), which can amplify ORF of β-actin were used. The PCR conditions were as follows: *HsPOLN, HsHAUS3,* β-actin, *MmPoln*, and *MmHaus3* −94 °C for 2 min, 30 cycles of 94 °C for 30 s, 55 °C for 45 s, 72 °C for 10 min, and 10-min extension at 72 °C; *MmRev3L* −94 °C for 2 min, 35 cycles of 94 °C for 30 s, 60 °C for 45 s, 72 °C for 1 min, and 5-min extension at 72 °C; *MmPolL* −94 °C for 5 min, 35 cycles of 94 °C for 1 min, 55 °C for 1 min, 72 °C for 10 min, and 10-min extension at 72 °C.

##### Cell Culture

293T and RKO cells were cultured in Dulbecco's modified Eagle's medium (DMEM) supplemented with 10% fetal bovine serum and 1% penicillin/streptomycin (Invitrogen). HeLa S3, 1618K, 833K, SuSa, and TERA1 cells were cultured in Roswell Park Memorial Institute (RPMI) 1640 medium supplemented with 10% fetal bovine serum and 1% penicillin/streptomycin. The cells were maintained in a humidified 5% CO_2_ incubator at 37 °C.

##### Plasmid Constructs

Full-length human POLN open reading frame (ORF) sequences were PCR-amplified as an XhoI-NotI fragment with 5′POLN (XhoI) primer (5′-CCGCTCGAGATGGAAAATTATGAGGCATTGGTAGGC-3′) and 3′POLN (NotI) primer (5′-TAAAAGCGGCCGCCTACAGACAAAATGAAGGCGAAAAATGC-3′) to clone into pOZN or pCDH-EF1-MCS-IRES-Puro, in which we inserted a V5 tag sequence to add the V5 tag at the N terminus of POLN. pCAM1224 plasmid carrying full-length wild-type BRCA1 ORF was kindly provided by Dr. Kevin Hiom. The BRCA1 ORF sequences were PCR-amplified as an XhoI-NotI fragment with 5′BRCA1 (XhoI) primer (5′- CCGCTCGAGATGGATTTATCTGCTCTTCGCGTTGAAG-3′) and 3′BRCA1 (NotI) primer (5′-TAAAAGCGGCCGCTCAGTAGTGGCTGTGGGGGATCTG-3′) to clone into pCDHN as follows: pCDH-EF1-MCS-IRES-Puro, in which we inserted the FLAG and HA tag sequence of pOZN to add the tag at the N terminus of POLN. Full-length wild-type FANCL ORF sequences were PCR-amplified as an XhoI-NotI fragment with 5′FANCL (XhoI) primer (5′-CCGCTCGAGATGGCGGTGACGGAAGCGAG-3′) and 3′FANCL (NotI) primer (5′-TAAAGCGGCCGCTCAGTGTTTCCTTCCAGAC-3′) and cloned into pOZN. Full-length wild-type FANCJ ORF sequences were PCR-amplified as an XhoI-NotI fragment with 5′FANCJ (XhoI) primer (5′-CCGCTCGAGTAAACCATGGCCTCTTCAATGTGGTCTGAATATAC-3′) and 3′ FANCJ (NotI) primer (5′-TAAAAGCGGCCGCCTTAAAACCAGGAAACATGCC-3′) and cloned into pCDHC, pCDH-EF1-MCS-IRES-Puro, in which we inserted the FLAG and HA tag sequence of pOZC to add the tag at the C terminus of *FANCJ*.

##### Affinity Purification of POLN and FANCL Complexes

HeLa S3 cells stably expressing FLAG-HA epitope-tagged POLN or FANCL were grown to 1.0 × 10^6^ cells/ml as 9 liters of suspension cultures (28, 29). The supernatant, nuclear extract, and chromatin fractions were prepared from the cells, and the POLN and FANCL complexes were immunoprecipitated from the nuclear extracts by incubating with M2 anti-FLAG-agarose gel for 4 h with rotation in the presence or absence of 50 units/ml Benzonase nuclease (Novagen). After an extensive wash with buffer 0.1B (100 mm KCl, 20 mm Tris-HCl, pH 8.0, 5 mm MgCl_2_, 10% glycerol, 1 mm PMSF, 0.1% Tween 20, 10 mm β-mercaptoethanol), the bound proteins were eluted from M2-agarose by incubation for 60 min with 0.2 mg/ml FLAG peptide (Sigma) in the same buffer. 100 μl of FLAG antibody-immunoprecipitated material was further purified by immunoprecipitation with anti-HA 12CA5 antibody conjugated to protein A-Sepharose (GE Healthcare). The bound proteins were washed with 0.1B and eluted with 17 ml of 0.1 m glycine HCl, pH 2.5. After the elution, the pH was neutralized with 3 μl of 1 m Tris-HCl, pH 8.0. To verify all proteins found in each complex by immunoblotting, only one gel was used per complex. Each membrane was cut horizontally into sections to immunoblot specifically for proteins of various sizes. Proteins were identified by LC-MS/MS using either the Proxeon Easy-nLC II or the Dionex Ultimate 3000 RSLCnano LC coupled to the Thermo Velos Pro or the Orbitrap Elite by analysis as reported in detail previously (13). Protein identification established greater than 99.9% protein probability assigned by the Protein Prophet algorithm, with a minimum of two peptides at 95% peptide probability. Peptide and protein false discovery rates were calculated as 0.0% by Scaffold. Abundant proteins commonly found in immunoprecipitation experiments with these epitope tags were eliminated from consideration (30–33). Protein identifications were checked for agreement with the molecular mass predicted from the relevant gel slice.

##### Immunoprecipitation

2.4 × 10^6^ 293T cells were plated in 10-cm plates 24 h prior to transfection. V5-POLN/pCDH or empty V5/pCDH was cotransfected with BRCA1/pCDHN, FANCJ/pCDHC, or empty pCDHC into the cells with Lipofectamine 2000 (Invitrogen). The cells were incubated with the plasmids for 24 h, washed, and replaced with fresh medium. 48 h after transfection, the cells were harvested, frozen in liquid nitrogen, and stored at −80 °C. Each cell pellet was suspended with 300 μl of buffer 0.5B (500 mm KCl, 20 mm Tris-HCl, pH 8.0, 5 mm MgCl_2_, 10% glycerol, 1 mm PMSF, 0.1% Tween 20, 10 mm β-mercaptoethanol), frozen in liquid nitrogen, thawed on ice, and sonicated in a 1.5-ml tube (1% amplitude, 5 s on, 15 s off for 9 cycles). After centrifugation, 900 μl of buffer 2B (40 mm Tris-HCl, pH 8.0, 20% glycerol, 0.4 mm EDTA, 0.2% Tween 20) was added to the supernatant and incubated with 10 ml of FLAG M2-agarose beads for 4 h at 4 °C in the presence or absence of 50 units/ml Benzonase nuclease. The bound proteins were washed with 700 μl of buffer 0.1B three times and eluted with 200 μl of 0.1B containing 0.5 mg/ml FLAG peptide. Eluted proteins were incubated with 30 μl of protein A-Sepharose conjugated with anti-HA 12CA5 antibody (GE Healthcare) for 4 h at 4 °C. Bound proteins were washed with 0.1B and eluted with 17 μl of 0.1 m glycine HCl, pH 2.5. After elution, pH was neutralized with 3 μl of 1 m Tris-HCl, pH 8.0.

##### Antibodies

Anti-FANCL (ab42639-100, 1:500 dilution) and anti-BRCA1 (ab16780, 1:1000 dilution) were purchased from Abcam. Anti-FANCA (A301-980A, 1:10,000 dilution) was purchased from Bethyl. Anti-FANCD2 (EPR2302, 1:2000 dilution) was purchased from GeneTex Inc. Anti-HELQ (1:1000 dilution) was from Cell Signaling Technology. Anti-α-tubulin (T5168, 1:8000 dilution), horseradish peroxidase (HRP)-conjugated anti-mouse IgG (A0168, 1:20,000 dilution), HRP conjugated anti-rabbit IgG (A0545, 1:20,000 dilution), anti-FLAG M2 (F3165), and anti-FLAG M2 affinity agarose gel (A2220) were purchased from Sigma. Anti-V5 antibody (R962-25) was purchased from Life Technologies, Inc. Anti-POLN (PA434) was raised against purified recombinant POLN (2, 3).

## Results

### 

#### 

##### Vertebrate DNA Polymerase ν

To extend studies of the expression and function of POLN, we isolated cDNA for *POLN* from the zebrafish *D. rerio POLN* (*DrPOLN*). Initial database searches found a genomic DNA sequence (NW_001879254) encoding several predicted exons homologous to human *POLN* (*HsPOLN*). Although most zebrafish cDNAs can be obtained from RNA of embryos, we were not able to amplify *POLN* from this source nor were we able to obtain *POLN* cDNA from pooled RNA extracted from whole adult fish. However, *POLN* cDNA sequences were readily recovered from RNA prepared from pooling the isolated testis regions of 25 adult fish. The entire *DrPOLN* cDNA was assembled by reverse transcriptase-PCR using 3′- and 5′-RACE-PCR techniques. This revealed that the zebrafish *POLN* genomic DNA includes 26 exons, encoding a protein of 1146 amino acids (sequence deposited as DQ630550). This is larger than human (900 amino acids, AAN52116) or mouse POLN (866 amino acids, AAN39837) (2). Protein alignment shows that DrPOLN has a longer intervening sequence between a short conserved N-terminal end region (designated POLN-N) and the DNA polymerase domain (Fig. 1*B*). The DNA polymerase domain of DrPOLN is 52.2% identical (71.7% similar) to HsPOLN and 56.7% identical (77.3% similar) to mouse POLN (MmPOLN), and all residues essential for DNA polymerase activity are conserved between predicted fish and mammalian POLN (Fig. 1*B*).

##### POLN and HAUS3 Transcripts Overlap, but Have Distinct Expression Patterns

**FIGURE 2.**
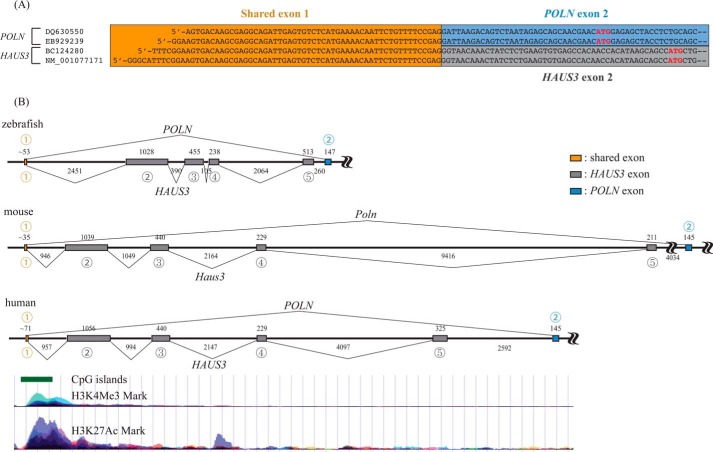
**Syntenic relationship between *POLN* and *HAUS3* genes in zebrafish, mouse, and human genomes.** *A,* sequence alignment of zebrafish *POLN* and *HAUS3* cDNA clones. *Orange, blue,* and *gray boxes* indicate the overlapping first exon of *POLN* and *HAUS3*, the second exon of *POLN,* and the second exon of *HAUS3*, respectively. The ATG start codon (*bold red letters*) resides within a well matched Kozak consensus sequence for translation initiation. DQ630550 is the GenBank^TM^ accession number for the sequence as determined by our experiments for *DrPOLN*; EB929239 is the GenBank^TM^ accession number for a cDNA clone encoding a partial *DrPOLN*. For *HAUS3*, BC124280 is the GenBank^TM^ accession number for cDNA encoding full-length *DrHAUS3*, and NM_001077171 is the NCBI reference sequence. *B,* zebrafish *POLN* and *HAUS3* genes are mapped on chromosome 7. Introns are represented as *black lines. Orange*, *gray*, and *blue boxes* denote the shared first exon, exons of *HAUS3,* and the second exon of *POLN*, respectively. Exons/introns are drawn to scale; each length (in bp) is shown. *Circled numbers* show the number of each exon. The first exon of *POLN* is also the first exon of *HAUS3* in mouse and human. Single major peaks of H3K27Ac and H3K4Me3, and a single CpG island are present near the first exon of human *POLN* and *HAUS3* but not before the second exon of *POLN* encoding the start codon. Data accessed from genome.ucsc.edu was derived from the ENCODE project.

**FIGURE 3.**
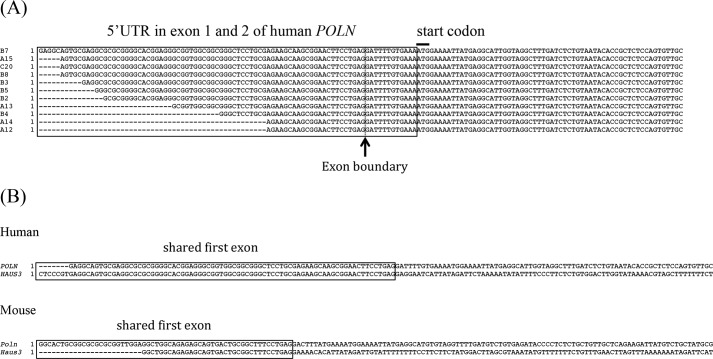
*A,* sequence alignment of 5′-RACE products for the human *POLN* cDNA. The newly identified 5′-untranslated region is shown within the *box*. A well matched Kozak consensus sequence for translation initiation surrounds the ATG start codon. *B,* sequence alignment of the first exon of human *POLN* (sequence of RACE-PCR clone B7 shown in *A*) and *HAUS3* mRNA (NCBI reference sequence, NM_024511.5), and mouse *Poln* (AY135562) and *Haus3* (NM_146159). Perfectly aligned sequences are *boxed*. The precise transcription initiation site is not known for *POLN* or *HAUS3* and could extend slightly 5′ of that shown for both mouse and human mRNAs.

Exons encoding the HAUS3 protein are located just upstream of and closely adjacent to *POLN* (Fig. 2). *HAUS3* encodes subunit 3 of the multisubunit augmin protein complex, critical for regulation of centrosome and spindle integrity. This syntenic relationship appears to be conserved in vertebrates. Surprisingly, our searches of transcript databases revealed that the zebrafish *POLN* transcript overlaps with the 5′UTR of *HAUS3*, indicating that both share the first exon (Fig. 2*A*). To investigate this further, we used RACE-PCR to determine the 5′UTR sequence of human *POLN*. All of the 5′UTR sequence clones for *HsPOLN* identified in this analysis overlapped with the 5′UTR of *HsHAUS3* (Fig. 3*A*). Similarly, the 5′UTR of the *Poln* transcript isolated from mouse testis RNA (2) overlaps with the 5′UTR sequence of *MmHaus3* (Fig. 3*B*). Thus for zebrafish, human, and mouse *POLN*, experimental data show that the coding exons for another gene (*HAUS3*) reside within the first intron (Fig. 2*B*), a unique arrangement among DNA polymerases.

**FIGURE 4.**
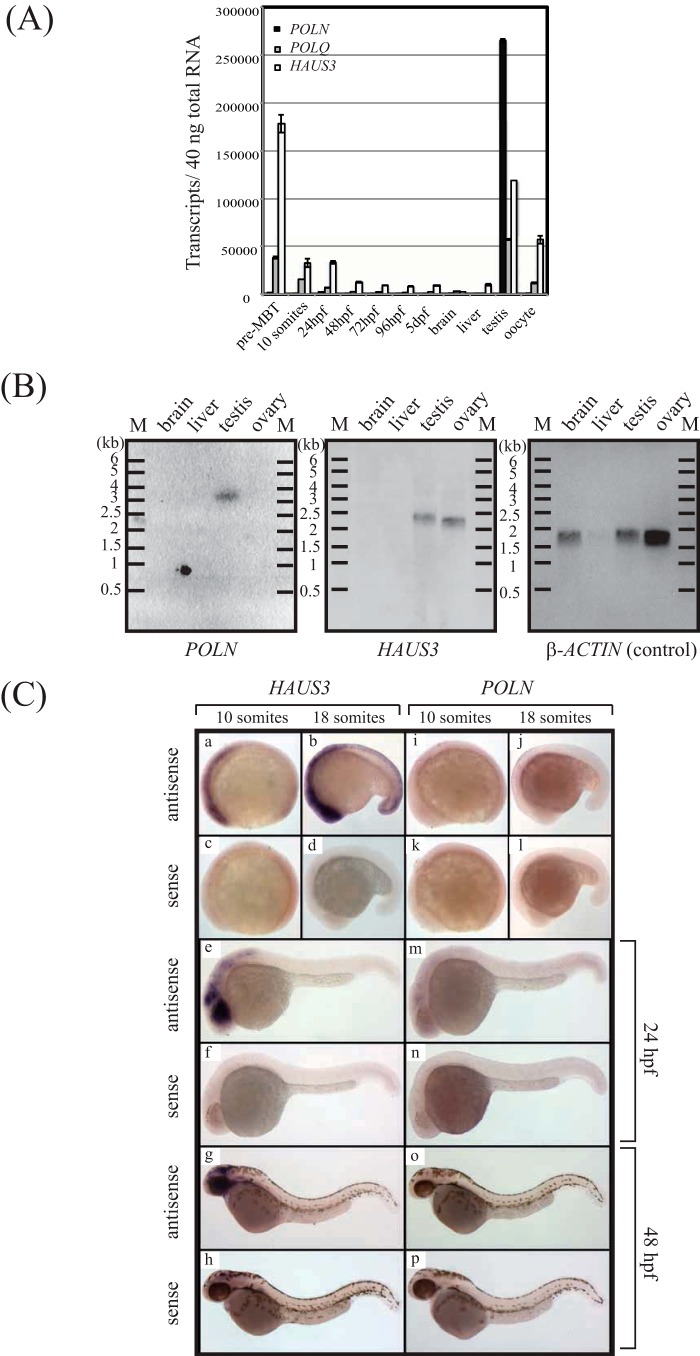
**HAUS3 but not *POLN* expression can be detected by *in situ* hybridization in zebrafish.** *A,* real time PCR analysis during zebrafish development. The *y* axis indicates transcripts per 40 ng of total RNA isolated from pre-mid-blastula transition (*Pre-MBT*) stage, 10-somite stage, 24-h post-fertilization (hpf) stage, and 48, 72, and 96-hpf, 5-day post-fertilization stage, brain, liver, testis, and oocyte (*B*). *POLN* expression in *D. rerio* is shown. Northern blot analysis in different adult tissues is shown. The same membrane was hybridized, stripped, and rehybridized sequentially with *D. rerio POLN, HAUS3,* or β-actin probes. *C,* embryonic expression patterns of zebrafish *POLN* and *HAUS3* mRNA. *Panels a–h, HAUS3 in situ* hybridization; *panels a, b, e,* and *g* are with the antisense probe, and *panels c, d, f,* and *h* are with the sense probe. *Panels i–p, POLN in situ* hybridization. *Panels i, j, m,* and *o* are with the antisense probe, and *panels k, l, n,* and *p* are with the sense probe. Stages: *panels a, c, i,* and *k* are 10 somites; *panels b, d, j,* and *l* are 18 somites; *panels e, f, m,* and *n* are 24 hpf; *panels g, h, o,* and *p* are 48 hpf.

In human cell lines, the pattern of histone H3K27 acetylation, H3K4 trimethylation, and a CpG island suggests a single promoter upstream of the shared first exon (ENCODE) (Fig. 2*C*) (34). However, the expression patterns of *POLN* and *HAUS3* are quite different, which can be explained by control at the level of alternative splicing of the primary transcript. In zebrafish, *POLN* is preferentially expressed in testis (Fig. 4*A*). In comparison, *HAUS3* and *POLQ* are expressed during embryonic development and in several tissues examined (Fig. 4, *A* and *B*). Consistent with this, *HAUS3* and *POLQ* are also broadly expressed in human tissues (Proteomic DB).

These observations were confirmed by *in situ* hybridization in zebrafish embryos. *HAUS3* transcripts were detected at all embryonic and larval stages analyzed, although *POLN* was not detectable by this technique (Fig. 4*C*). We conclude that *POLN* is expressed weakly or not at all in zebrafish embryos, which agrees with our inability to recover the *POLN* mRNA from embryos by RT-PCR. We injected a *POLN*-specific antisense MO into fish embryos. Eighty three, 62, and 22 embryos were injected with 2.5, 5, and 10 ng of *POLN* MO, respectively. Thirty two control embryos were mock-injected. However, no alteration in development was detected with either *POLN*-specific or control MO. This suggests that POLN does not have an essential function in early development, consistent with the lack of expression in the embryo.

**FIGURE 5.**
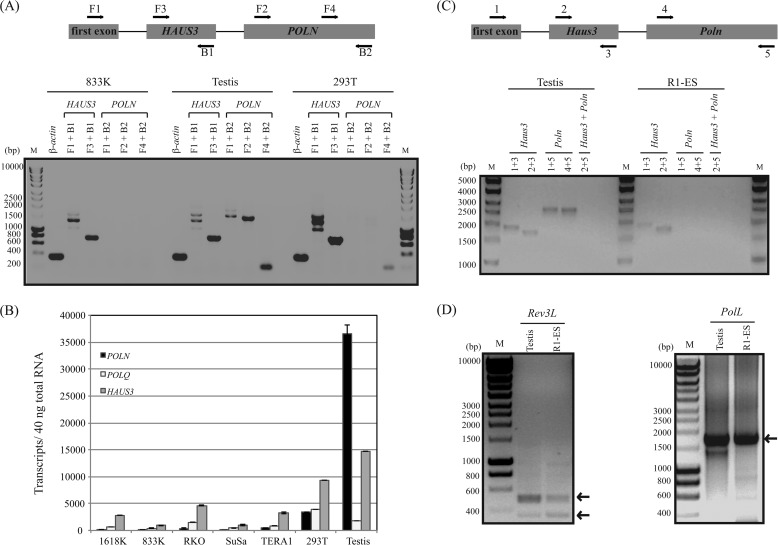
**Independent expression of *POLN* and *HAUS3* and preferential expression of *POLN* in testis.** *A,* RT-PCR analysis of *POLN* and *HAUS3* in 833K, human testis, and 293T cDNA. PCR was performed with primers in exon 1 of *POLN* and *HAUS3* (*F1*), ORF of *HAUS3* (*F3* and *B1*), and ORF of *POLN* (*F2*, *F4*, and *B2*). Positions of the primers are diagramed above the gel picture. β-Actin was used as a control. Expected product size (bp) are F1 + B2, 1474; F2 + B2, 1384; F4 + B2, 203; F1 + B1, 999; F3 + B1, 736; β-actin, 353. *B,* real time PCR analysis in human cultured cells and human testis. The *y* axis indicates the absolute quantity of transcripts for *POLN*, *POLQ,* and *HAUS3* per 40 ng of total RNA isolated from 1618K, 833K, RKO, SUSA, TERA1, 293T, and testis. *C,* RT-PCR analysis of *Poln* and *Haus3* in mouse testis and R1-ES cDNA. PCR was performed with primers in exon 1 of *Poln* and *Haus3* (*1*), ORF of *Haus3* (*2* and *3*), and ORF of *Poln* (*4* and *5*). Positions of the primers are diagramed *above* the gel picture. *D, Rev3L* and *PolL* were used as controls. Expected product size (bp) are 1 + 3, 1867; 2 + 3, 1713; 1 + 5, 2628; 4 + 5, 2595; *Rev3L,* 350 and 478; *PolL*, 1720. PCR products were separated on a 1% agarose gel and visualized with EZ-Vision DNA Dye (*A*) or ethidium bromide (*C* and *D*).

The expression patterns of *POLN* and *HAUS3* were also distinct in mammals. We compared expression of *POLN*, *POLQ,* and *HAUS3* in mRNA from human testis and human cell lines (Fig. 5). All three genes were readily detected in testis. In cultured cells, only low levels of partial *POLN* transcript were detectable by RT-PCR (Fig. 5*A*) and by real time PCR (qPCR) (Fig. 5*B*). Using RT-PCR, we designed primers to test whether *POLN* and *HAUS3* are independent mature spliced transcripts. The F1-B2 primer pair amplified a product from human testis of the size expected for *POLN* but not for a fusion of *HAUS3* and *POLN* transcript (Fig. 5*A*). Consistently, no evidence for a fused transcript was found in the 5′-RACE experiments (Fig. 3*A*). Full-length *POLN* transcript was not detected by this primer set in the human 833K or 293T cell lines, although a transcript representing a portion of the mRNA could be detected (primer set F4 + B2). The F1 + B1 primer set yielded two major bands and one minor band. The major bands are consistent with the predicted size of the two documented transcript variants of human *HAUS3* (accession numbers NM_001303143 and NM_024511). A third lower molecular weight band arising with this primer set may also be an alternatively spliced product.

We also examined expression of *Haus3* and mRNA encoding DNA polymerases in mouse testis and embryonic stem (ES) cells. Transcripts for *Haus3*, *Poln*, *Rev3L* (the catalytic subunit of DNA polymerase ζ), and *PolL* (DNA polymerase λ) were readily detected in mouse testis (Fig. 5, *C* and *D*). In contrast, mouse ES cells expressed *Haus3*, *Rev3L,* and *PolL,* whereas *Poln* was not detected (primers 1 + 5 and 4 + 5, Fig. 5*C*). As found with human cells, no transcript fusing both *Haus3* and *Poln* was detectable (primers 2 + 5, Fig. 5*C*).

##### Zebrafish POLN Retains Strand Displacement and Bypass Activities

**FIGURE 6.**
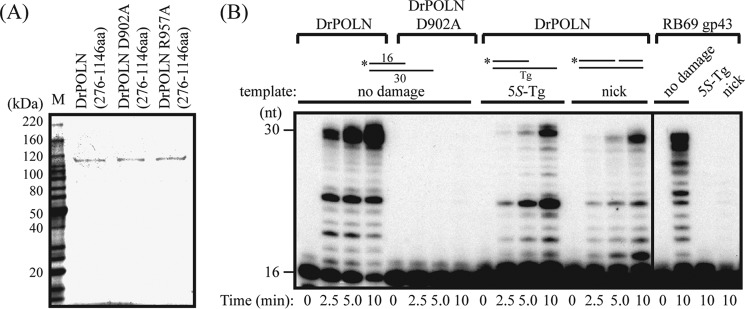
**DNA polymerase activity of zebrafish POLN (DrPOLN).** *A,* constructs containing residues 276–1146 of DrPOLN were bacterially expressed and purified. Substituted residues in DrPOLN derivatives (Asp-902 and Arg-957) are shown in Fig. 1. Three hundred ng of purified DrPOLN derivatives and molecular mass markers were separated by electrophoresis in a 4–15% SDS-polyacrylamide gradient gel and stained with colloidal Coomassie Brilliant Blue G-250. *B,* DNA polymerase activities of DrPOLN. 23 nm DrPOLN and DrPOLN (D902A) and 10 pm RB69 gp43 were incubated with the 5′-^32^P-labeled primer-templates indicated under “Experimental Procedures” in the presence of all four dNTPs at 37 °C for the indicated time. The activities were analyzed on the same gel.

**FIGURE 7.**
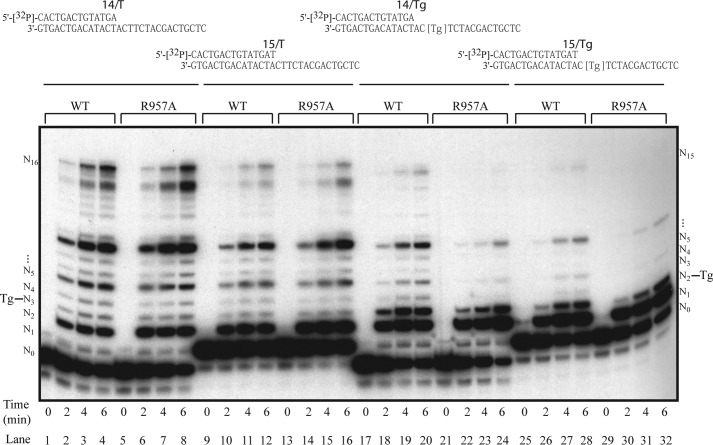
**Evolutionarily conserved residue is important for translesion synthesis activity in DrPOLN.** *A,* 23 nm DrPOLN and DrPOLN (R957A), denoted as WT and R957A, respectively, were incubated with the 5′-^32^P-labeled primer-templates indicated *above* the panel; DNA synthesis on a DNA template containing an undamaged thymine (*lanes 1–16*) or a 5*S*-Tg (*lanes 17–32*) from the 14-mer primer (*lanes 1–8* and *17–24*) or the 15-mer primer (*lanes 9–16* and *25–32*) is shown. All reaction mixtures contained substrate at 100 nm in the presence of all four deoxynucleotide triphosphates. Incubation time of each reaction is shown at *bottom*. Locations of unreacted end-labeled primer (*N*_0_), each template base position (from *N*_1_ to *N*_16_), full-length product (*N*_16_ for the 14-mer primer and *N*_15_ for the 15-mer primer), and positions of 5*S*-Tg are shown as *Tg*.

The *DrPOLN* cDNA encoding amino acids 276–1146 was expressed in *E. coli*, tagged with six His residues at the N terminus, and a FLAG epitope tag at the C terminus. An active site mutant (D902A) and a mutant with a substitution in an evolutionarily conserved residue of POLN (R957A) were also expressed (Fig. 6*A*). Asp-902 corresponds to a highly conserved residue in motif 3 of A-family DNA polymerases and is important in coordinating bivalent metal ions to interact with an incoming deoxynucleotide triphosphate (Fig. 1*B*) (3, 35). Arg-957 corresponds to the Lys-679 of human POLN, important for bypass activity and fidelity (Fig. 1*B*) (4, 5). Proteins sequentially purified on FLAG antibody beads and metal affinity resin migrated near the expected molecular mass of 104 kDa (Fig. 6*A*). DrPOLN was able to extend DNA on a primed template, although the active site mutation (D902A) abrogated its DNA polymerase activity (Fig. 6*B*). No exonuclease activity was detected when enzyme and substrate were incubated without dNTPs, consistent with the lack of critical conserved residues for 3′–5′-exonuclease activity (Fig. 1*B*) (36), as found with HsPOLN. DrPOLN efficiently bypassed a (5*S*)-thymine glycol (5*S*-Tg) in DNA. Quantification (Fig. 7 and Table 2) showed that the Tg bypass efficiency of DrPOLN was 18.0%, similar to the 15.8% observed for human POLN (4). Like HsPOLN, DrPOLN also showed efficient strand displacement activity on a nicked DNA substrate (Fig. 6*B*). For comparison, the DNA polymerase RB69 gp43 did not show these activities on the same substrates (Fig. 6*B*).

**TABLE 2:** **Bypass, insertion, extension probabilities and bypass efficiency** The bypass probability at position *N* is defined as the band density ≥(*N* + 1) divided by the intensity of ≥(*N*_1_). The insertion probability at any position (*N*) is defined as the intensity at bands ≥(*N*) divided by the intensity at bands ≥(*N* − 1). The extension probability at any position (*N*) is defined as the band intensity ≥(*N* + 1) divided by the intensity at bands ≥(*N*). To detect the bypass efficiency, the bypass probability (damaged) is divided by the bypass probability (undamaged) (25).

Enzyme	Template	Bypass probability	Insertion probability	Extension probability	Bypass efficiency
Wild type	14/T	67.8 ± 0.9	72.9 ± 0.3	95.5 ± 0.1	
R957A	14/T	63.6 ± 0.7	67.5 ± 0.5	95.9 ± 0.1	
Wild type	14/Tg	12.2 ± 0.2	29.6 ± 0.5	41.8 ± 0.2	18.0 ± 0.5
R957A	14/Tg	2.4 ± 0.2	13.0 ± 0.1	19.0 ± 1.3	3.8 ± 0.3
Wild type	15/T	44.6 ± 0.2	49.2 ± 0.2	90.8 ± 0.7	
R957A	15/T	41.9 ± 0.7	45.7 ± 0.4	91.7 ± 0.8	
Wild type	15/Tg	4.5 ± 0.2	15.1 ± 0.8	29.8 ± 3.1	10.1 ± 0.5
R957A	15/Tg	1.4 ± 0.1	10.9 ± 2.1	13.2 ± 1.8	3.4 ± 0.3

A conserved Lys or Arg residue (Lys-679 in HsPOLN and Arg-957 in DrPOLN) was identified in the “O-helix” of motif 4 in the finger subdomain (Fig. 1*B*) (4). The corresponding residue is one of the most important for controlling fidelity of prokaryotic polymerase I and is a nonpolar Ala or Thr in those enzymes (37, 38). The residue was important for low fidelity and bypass activity in HsPOLN. K679A or K679T HsPOLN (polymerase I-like) mutants showed higher fidelity than wild-type HsPOLN but did not bypass a 5*S*-Tg efficiently. A K679R HsPOLN (DrPOLN-like) mutant bypassed a 5*S*-Tg as efficiently as wild-type HsPOLN (4). We examined the corresponding residue in zebrafish POLN. The R957A mutation reduced 5*S*-Tg bypass efficiency by 5-fold, without significantly affecting DNA polymerase activity on undamaged DNA (Fig. 7). Thus, a basic residue at this position is important for translesion synthesis activity of POLN.

**FIGURE 8.**
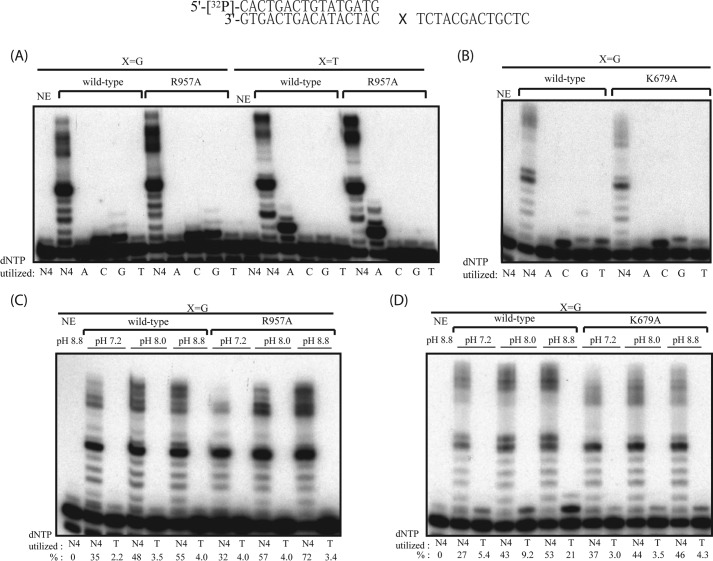
**Nucleotide selectivities of DrPOLN and HsPOLN derivatives.** *A,* 23 nm DrPOLN and DrPOLN (R957A), denoted as wild-type and R957A, respectively, were incubated with 300 fmol of 5′-^32^P-labeled 16-mer primer annealed to a 30-mer DNA template in the presence of four or one of the indicated dNTPs (100 μm) for 10 min. The first template base denoted by *X* was G or T. Template sequences are indicated *above* the panel. *NE* indicates no enzyme. *B,* as described for *A*, using HsPOLN and HsPOLN (K679A), denoted as wild-type and K679A, respectively. *C,* 23 nm DrPOLN and DrPOLN (R957A), denoted as wild-type and R957A, respectively, were incubated with 5′-^32^P-labeled 16-mer primer annealed to a 30-mer DNA template, in which the first template base was G in the presence of all four dNTPs or dTTP (100 μm) for 10 min in indicated pH conditions. *D,* as described for *C*, using HsPOLN and HsPOLN (K679A), denoted as wild-type and K679A, respectively. The percentage (%) of the product extension from the primer is shown *below* each lane in *C* and *D*.

Low fidelity favoring incorporation of T for template G is a biochemical property of HsPOLN (3–5). However, this tendency was not evident in DrPOLN (Fig. 8). Wild-type HsPOLN but not the K679A or K679T HsPOLN mutants efficiently incorporated T for G at the optimal pH of 8.8 (3). When the pH was reduced to 8.0 or 7.2, the DNA polymerase activity and the T misincorporation activity of HsPOLN were both reduced as reported (Fig. 8, *B* and *D*) (23). Unlike HsPOLN, wild-type DrPOLN did not efficiently incorporate T opposite template G even at pH 8.8; the Arg residue at 957 did not influence T or G misincorporation (Fig. 8, *A* and *C*). In addition, DrPOLN efficiently incorporated G opposite G unlike HsPOLN (Fig. 8, *A* and *B*).

##### POLN Protein Associations

**FIGURE 9.**
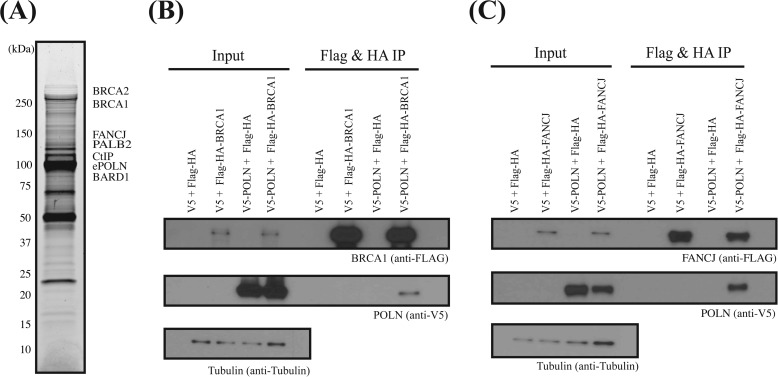
**POLN is associated with BRCA1, FANCJ, and other homologous recombination components.** *A,* POLN-associated proteins were immunopurified from nuclear extracts prepared from HeLa S3 cells expressing FLAG-HA epitope-tagged POLN. The complex was sequentially purified with anti-FLAG and anti-HA antibodies, resolved by SDS-PAGE on a 4–20% gradient gel, and visualized by silver staining. Approximate migration positions of proteins identified in gel sections are shown. *B,* V5-tagged POLN and FLAG-HA epitope-tagged BRCA1 were transiently co-expressed in 293T cells. The whole-cell extracts prepared from the transfected cells were sonicated and incubated in the presence of benzonase. FLAG-HA epitope-tagged BRCA1 was immunoprecipitated (*IP*) with anti-FLAG and anti-HA antibodies. V5-tagged POLN in the immunoprecipitated samples was detected with anti-V5 antibody. *C,* interaction between V5-tagged POLN and FLAG-HA epitope-tagged FANCJ was examined similarly as described in *B*.

**FIGURE 10.**
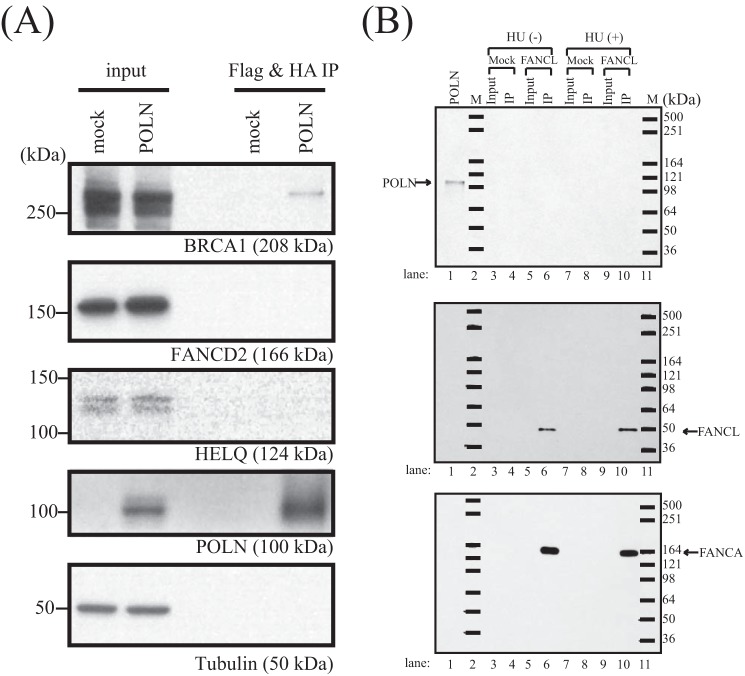
*A,* POLN complex was immunopurified from nuclear extract prepared from HeLa S3 cells expressing FLAG-HA epitope-tagged POLN. Immunoblotting with specific antibodies confirmed the presence or absence of the proteins in the POLN complex. A single membrane was cut into sections as shown in supplemental Fig. S1 for immunoblotting with different antibodies. One of the membrane sections was first used for HELQ immunoblotting and then stripped using Thermo Restore Western Blot Stripping Buffer and reblotted for identification of FANCD2 and then again for POLN. *B,* POLN does not interact with the Fanconi core complex in the presence or absence of HU. The FANCL complex was immunopurified from nuclear extracts prepared from HeLa S3 cells expressing FLAG-HA epitope-tagged FANCL with or without 3 mm HU treatment for 24 h. The complex was sequentially purified with anti-FLAG and anti-HA antibodies. The complex was resolved by SDS-PAGE on a 4–20% gradient gel. Immunoblotting with specific antibodies confirmed the presence or absence of the proteins in the FANCL complex. FANCA but not POLN is present in the complex before and after HU treatment. Crude extract prepared from 293T cells transiently expressing POLN (lane labeled *POLN*) was used as a positive control for the POLN antibody.

It has been suggested that POLN interacts with several proteins, including HELQ, Fanconi anemia (FA) core complex proteins, and FANCD2 (16). It is intriguing that this set of proteins has testis-related functions. *Helq* knock-out male mice have significantly smaller testes (14), targeted disruption of several FA genes caused impaired fertility in mice (39), and infertility is a common feature among male FA patients (40). To test these proposed interactions and to uncover possible molecular pathways relevant to POLN, we searched for proteins with the potential to associate with POLN *in vivo*. POLN was stably expressed as a FLAG and HA epitope fusion (ePOLN) in HeLa S3 cells. ePOLN was recovered from nuclear extracts by sequential immunoprecipitation with anti-FLAG and anti-HA antibodies (28, 29). The immunoprecipitate was separated on a gradient gel (Fig. 9*A*), and proteins from gel sections were identified by liquid chromatography-mass spectrometry. The data were filtered to eliminate common s. Among the resulting top 150 ranking hits there were nine DNA repair-related proteins (supplemental Table 1). Six of these (BRCA1, BRCA2, BARD1, PALB2, FANCJ, and RBBP8/CtIP) are components of the A, B, and C BRCA1-associated complexes related to homologous recombination (Fig. 9*A* and Table 3) (41). None of these proteins were present in a control FLAG-HA purification from HeLa S3 cells transfected with empty control vector. In parallel experiments in the same system using FLAG-HA-tagged HELQ as bait, none of these proteins were detected in the HELQ complex (13). Although FA core complex proteins FANCD2 and HELQ were previously proposed as POLN-interacting partners (16), no peptides representing any of these proteins were identified as ePOLN-associated proteins (supplemental Table 1), just as POLN was not detected in the HELQ complex in the previous study (13). Immunoblotting confirmed that BRCA1 was present in the immunoprecipitate and that FANCD2 and HELQ were present in the input fraction but undetectable in the POLN immunoprecipitate (Fig. 10*A*). To confirm POLN-BRCA1 and POLN-FANCJ interactions in another cell line, V5-tagged POLN was coexpressed with FLAG-HA-tagged BRCA1 or FANCJ in 293T cells. After immunoprecipitation from whole-cell extract with anti-FLAG and anti-HA antibodies, V5 antibody was used to identify POLN in the immunoprecipitate. Co-immunoprecipitation was observed between POLN and BRCA1 (Fig. 9*B*) and POLN and FANCJ (Fig. 9*C*).

**TABLE 3:** **Recombination proteins identified in the POLN complex by LC-MS/MS analysis** Proteins from gel sections were identified by LC-MS/MS. The gel sections were approximately equal size. No peptides were detected that matched HELQ or proteins in the FA core complex.

Protein	Accession no. (UniprotKB/Swiss-Prot)	Molecular mass	Spectral counts	Unique peptides
		*kDa*		
POLN	Q7Z5Q5|DPOLN_HUMAN	100	1207	349
BRCA1	P38398|BRCA1_HUMAN	208	23	15
BARD1	Q99728|BARD1_HUMAN	87	15	8
PALB2	Q86YC2|PALB2_HUMAN	131	17	11
BRCA2	P51587|BRCA2_HUMAN	384	7	7
FANCJ	Q9BX63|FANCJ_HUMAN	141	16	12
CtIP	Q99708|COM1_HUMAN	102	15	8

We also tested for an association of the FA core complex with POLN. The same system was employed but with FANCL as bait. FANCL was stably expressed as a FLAG and HA epitope fusion (eFANCL) in HeLa S3 cells, with or without exposure to hydroxyurea (HU). After sequential immunoprecipitation with anti-FLAG and anti-HA antibodies, an FA core complex protein, FANCL, was identified in the immunoprecipitate, as expected (Fig. 10*B*). However, POLN was not present, consistent with its normally low or absent expression in human cultured cells.

## Discussion

These investigations by biochemical, molecular genetic, and proteomic analysis answer several outstanding questions about vertebrate POLN.

First, the expression of *POLN* in vertebrate cells is very limited and tissue-specific. In zebrafish, mouse, and humans, *POLN* is preferentially expressed in testis and very weakly in other tissues or cells in culture. *POLN* was originally assembled by analyzing transcripts and expressed sequence tags from human cell lines (2). However, only short fragments of *POLN* are readily isolated by RT-PCR from cDNA of human cell lines (Fig. 5*A*). RACE-PCR was used to clone the 5′UTR sequence of human *POLN* from testis cDNA (Fig. 3*A*) without difficulty, but we were unable to do this from human cell lines. In the human cell lines examined so far, POLN is subject to extensive alternative splicing that gives rise to biologically inactive transcripts (2). This is important to consider when evaluating data from microarrays or RNA-sequence experiments, as most *POLN* mRNA expression will represent biologically inactive transcripts.

Second, we report new evidence that the *POLN* and *HAUS3* genes share the same first exon, in an evolutionarily conserved manner. From analysis of mouse database annotations, it was suggested that *HAUS3* and *POLN* do not overlap (42), but our primary analysis of sequences from three vertebrates indicates that *POLN* and *HAUS3* share a single promoter and first exon. Peaks of H3K27 acetylation and H3K4 trimethylation, and a CpG island, which are often found near active regulatory elements, were identified near the first exon of *POLN* and *HAUS3* but not around the other exons. *POLN* and *HAUS3* may initiate transcription from the same promoter, with their expression regulated by tissue/cell type-specific splicing of the transcripts. A recent study underlines that modulation of splicing can indeed influence *POLN* mRNA expression levels (43). Small molecule compounds were identified that shifted RNA splicing of the *SMN2* gene toward production of full-length mRNA. Intriguingly, *POLN* was one of only six genes in human cells that increased in expression by a factor of >2 after treatment with one of the most specific small molecule compounds, SMN-C3 (43). In a large family of genes involved in DNA or RNA metabolism, *POLN* was the only such regulated gene. POLN is unique among mammalian DNA polymerases in its conserved expression with an overlapping gene. The possible selective advantage for this arrangement is not known, but it is notable that it shares a promoter region with a ubiquitously expressed housekeeping gene (*HAUS3*). This might provide a mechanism for a small amount of POLN to be produced by alternative splicing in any cell type, when POLN is needed for a specialist function. RNA transcript splicing patterns can vary considerably in different tissues (44), which could allow large amounts of POLN to be produced in specific situations when necessary, as appears to be the case in testis. It remains to be seen whether the levels of *HAUS3* are also subject to splicing modulation. This could be relevant as *HAUS3* is sometimes mutated in breast cancer. In a study following a lobular human breast tumor, a mutation in *HAUS3* was one of only five nonsynonymous coding mutations that were prevalent in the primary breast tumor and remained in the metastatic cancer nine years later (45).

Third, it was not known whether *POLN* is an essential gene for embryonic development. We found that it is not essential in zebrafish. This is because *POLN* is not appreciably expressed in zebrafish embryos, and because no phenotypic differences were identified after the injection of control and *POLN*-specific antisense morpholino oligonucleotides.

One possibility is that POLN may have a function in testis, where the gene is preferentially expressed. Morpholino antisense oligonucleotides can be used to analyze phenotypes in early developmental stages (46) but not readily in adult testis. In human cells, *POLN* is also highly expressed in testis but very weakly expressed in cell lines, even those derived from testicular cancers (1618K, 833K, SuSa, and TERA1). Here, we identified the potential for POLN to interact in a protein complex with homologous recombination-related proteins, including BRCA1 and downstream FA proteins, including FANCJ, BRCA2 (FANCD1), and PALB2 (FANCN). This is consistent with a possible function of POLN in homologous recombination in testis. However, our results do not support the previously proposed interactions of POLN with HELQ, FA core complex proteins, or FANCD2. During the process of meiotic recombination, the evolutionarily conserved strand displacement activity of POLN could be useful to synthesize DNA in D-loop recombination intermediates. Another possible function is chromatin remodeling in the XY body of spermatocytes, because BRCA1 has a role in the establishment of X-pericentric heterochromatin in testis (47).

Finally, we found that the ability to bypass thymine glycol lesions in DNA is a property conserved in the human and zebrafish enzymes and that a basic residue in the O-helix (human residue Lys-679) is crucial for this activity in both species. Tg is a major DNA lesion generated by reactive oxygen species that blocks the progression of replicative DNA polymerases (48). However, we do not yet know whether the ability of POLN to bypass Tg lesions is physiologically relevant (for example, in a testis-specific role). It is possible that the bypass activity is only an *in vitro* readout of the unusual active site of POLN, which normally functions in some other challenging role, such as strand displacement. One of the evolutionarily conserved sequence insertions in the DNA polymerase domain of POLN (4), called insert 2, forms a unique cavity in the DNA polymerase domain of POLN (5). The cavity allows POLN to generate and accommodate a looped-out primer strand (5), and it may also help POLN to bypass a Tg lesion by tolerating the distortion generated after incorporation of A opposite Tg (48). Similarly, human POLN is striking in its very high G to A base substitution rate. We found no indication of a marked tendency for zebrafish POLN to incorporate T opposite template G. In steady-state conditions, human POLN preferentially incorporates T opposite G but not in the pre-steady-state (21). The low fidelity of human POLN might be a result of assaying the enzyme at its optimum pH for activity, pH 8.8 (3). Other A-family DNA polymerases also show increased fidelity at lower pH (49, 50). It remains to be determined whether nucleotide misincorporation is relevant to an *in vivo* function of POLN.

## Author Contributions

K. T. and R. D. W. conceived and designed the experiments. K. T., J. T., S. R., and L. M. A. performed the experiment. K. T., J. T., S. R., L. M. A., M. T., N. A. H., and R. D. W. analyzed the data. K. T. and R. D. W. wrote the paper.

## Supplementary Material

Supplemental Data

## Footnotes

3 The abbreviations used are:

Tg: thymine glycol
RACE: rapid amplification of cDNA ends
MO: morpholino oligonucleotide
qPCR: quantitative PCR
HU: hydroxyurea
FA: Fanconi anemia
hpf: h post-fertilization.
