# Supplementary material for: Conserved Overlapping Gene Arrangement, Restricted Expression, and Biochemical Activities of DNA Polymerase ν (POLN)
Source: J Biol Chem. 2015 Aug 12;290(40):24278–93. doi: 10.1074/jbc.M115.677419 (PMC4591814; doi:10.1074/jbc.M115.677419)
Supplement: Supplemental Data [file supp_290_40_24278__index.html]

Conserved overlapping gene arrangement, restricted expression and biochemical activities of DNA polymerase ν; (POLN) — Conserved Overlapping Gene Arrangement, Restricted Expression, and Biochemical Activities of DNA Polymerase ν (POLN) — POLN and HAUS3 Share First Exon — Supplemental Data 

# Conserved Overlapping Gene Arrangement, Restricted Expression, and Biochemical Activities of DNA Polymerase ν (POLN)

## Supplemental Data

- Supplemental Table 1 (.xlsx, 38 KB) - Top 150 ranking hits in the POLN complex
